# Adnexal Torsion Associated With Recurrent Ovarian Hyperstimulation Syndrome: A Case Report

**DOI:** 10.7759/cureus.36344

**Published:** 2023-03-19

**Authors:** Nafeesathu Misiriyyah, Khansa Qurban, Aasiya Beevi, Litty Paulose, Haroutyoun Margossian

**Affiliations:** 1 Department of Medicine, Dubai Academic Health Corporation, Dubai, ARE; 2 Department of Medicine, Dubai Medical College, Dubai, ARE; 3 Department of Medical Education, Dubai Medical College for Girls, Dubai, ARE; 4 Department of Obstetrics and Gynecology, Latifa Women and Children Hospital, Dubai, ARE

**Keywords:** detorsion, cyst aspiration, adnexal torsion, ivf, recurrent ovarian hyperstimulation syndrome

## Abstract

Adnexal torsion due to hyperstimulation is one of the well-recognized serious complications of assisted reproductive techniques like in vitro fertilization (IVF). We report a case of a 35-year-old primigravida who presented to the emergency department with complaints of acute severe left iliac fossa pain and nausea. Further history revealed that the patient had previously undergone one cycle of ovulation induction and was diagnosed with ovarian hyperstimulation syndrome (OHSS). After prompt management, she reportedly underwent a cycle of IVF successfully. Her gestational age was five weeks and one day on presentation. A transabdominal ultrasound revealed bilateral enlarged ovaries with adequate blood supply and a single intrauterine gestational sac with a yolk sac.

The patient was admitted as a case of recurrent OHSS for conservative management. Despite the initial improvement of symptoms with parenteral analgesia, an emergency laparoscopic surgery was done later due to worsening symptoms. Intraoperative findings were consistent with the suspected diagnosis of adnexal torsion which was managed accordingly. Postoperatively, the patient recovered without complications and was discharged two days later with a plan for outpatient follow-up.

## Introduction

Adnexal torsion associated with ovarian hyperstimulation syndrome (OHSS) is an uncommon, potentially life-threatening condition with a prevalence of 0.02% in pregnancy [1]. The exact pathophysiology of this condition is unclear but is hypothesized to be related to human chorionic gonadotropin (hCG) mediated ovarian hypersensitivity and enlargement, which can predispose to adnexal torsion [2]. Due to several pregnancy-related factors, torsion is often misdiagnosed as other acute abdominal conditions [3]. Surgical exploration is considered the gold standard for diagnosis [4]. The conventional treatment modality of salpingo-oophorectomy for ovarian torsion is widely being replaced by the ovary-sparing option of detorsion [5].

## Case presentation

A 35-year-old primigravida was treated with one cycle of in vitro fertilization (IVF) for primary infertility of one and a half years. She reportedly received ovarian stimulation therapy with gonadotropins followed by oocyte cryopreservation five months ago. She was subsequently diagnosed with bilateral ovarian enlargement due to hyperstimulation which was managed with a high-protein diet and cabergoline. She reportedly recovered completely as evidenced by an ultrasound scan done before embryo transfer.

Her last menstrual period was seven weeks and one day prior to the presentation. A frozen embryo transfer was done on day 29 of her last menstrual cycle. Her pregnancy was confirmed with serum beta-human chorionic gonadotropin (hCG) levels two weeks later. She received three doses of hCG in the same cycle and continued aspirin, estrogen, and progesterone.

The patient was on a transatlantic trip when she developed severe acute spasmodic abdominal pain in the left iliac fossa and nausea at the transit airport. She was transferred to a women’s specialty hospital after the administration of parenteral analgesia.

Physical examination revealed a vitally stable, mildly dehydrated female in distress due to pain. Her body mass index (BMI) was 16.8 kg/m². Her abdomen was soft and lax with severe localized tenderness, guarding, and a palpable smooth globular mass measuring 8 cm in the left iliac fossa which was dull on percussion. Examination of all other systems was unremarkable. Lab investigations revealed serum beta-hCG levels consistent with her gestational age, elevated white blood cell (WBC) count, and low potassium levels. All biochemical tests obtained on admission are summarized in Table 1.

**Table 1:** Laboratory studies obtained on admission. WBC: white blood cell; RBC: red blood cell; pH: potential of hydrogen; pCO_2_: partial pressure of carbon dioxide; pO_2_: partial pressure of oxygen; FHbF: fraction of hemoglobin F; PCR: polymerase chain reaction; hCG: human chorionic gonadotropin

Laboratory test		Laboratory value	Normal reference range
Complete blood count	WBC count	17.1 k/uL	3.6-11.0 k/uL
	RBC count	4.08 Mil/uL	3.80-4.80 Mil/uL
	Hemoglobin	12.2 g/dL	12.0-15.0 g/dL
	Hematocrit	36.2%	36.0-46.0%
	Platelets count	391 k/uL	150-410 k/uL
Venous blood gas, metabolic panel	pH	7.417	7.35-7.45
	pCO_2_	31.0 mmHg	40-50 mmHg
	pO_2_	28.1 mmHg	30-55 mmHg
	FHbF	21%	0-2%
General biochemistry	Sodium, serum	134 mmol/L	136-145 mmol/L
	Potassium, serum	2.9 mmol/L	3.3-4.8 mmol/L
	Bicarbonate, serum	18.9 mmol/L	20-28 mmol/L
	Urea, serum	11 mmol/L	12-40 mmol/L
	Ionized calcium, serum	1.13 mmol/L	1.16-1.31 mmol/L
	Glucose, serum	87 mg/dL	60-100 mg/dL
	Lactic acid, serum	1.3 mmol/L	0.5-2.2 mmol/L
	Creatinine, serum	0.5 mg/dL	0.5-0.9 mg/dL
	Bilirubin, serum total	0.3 mg/dL	0-1.2 mg/dL
	Alkaline phosphatase, serum	32 U/L	35-104 U/L
	Alanine aminotransferase, serum	06 U/L	0-31 U/L
	Total protein, serum	7.3 g/dL	6.6-8.7 g/dL
	Albumin, serum	4.2 g/dL	3.4-4.8 g/dL
	Fibrinogen, serum	442 mg/dL	190-430 mg/dL
	Prothrombin time	11.7 secs	11.5-14.5 secs
	Activated partial thromboplastin time	46.9 secs	28.6-38.2 secs
	Total beta-hCG, serum	9177 mIU/mL	1159 -46,782 mIU/mL (5 weeks gestation)
	Progesterone, serum	131 nmol/L	35-141 nmol/L (first trimester)
	SARS-CoV-2 RNA PCR-SWAB nasopharynx	Not detected	Not detected
	Blood group	A positive	-

**Figure 1:**
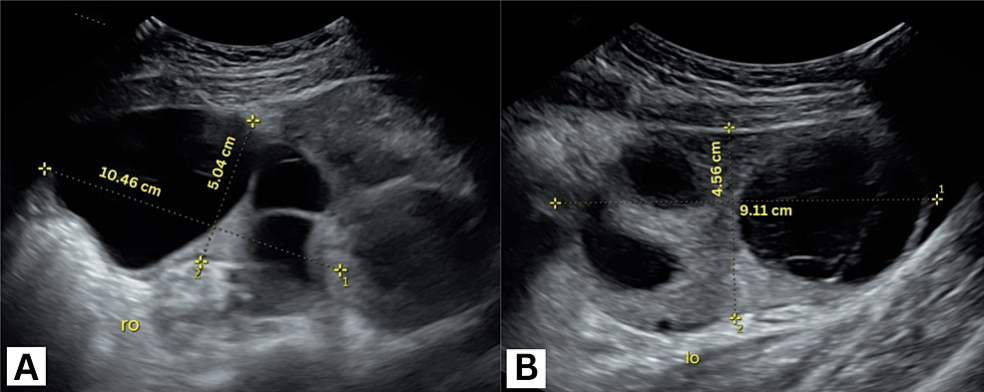
Transabdominal ultrasound images showing bilateral enlarged ovaries with multiple cysts; (A) right ovary (ro) and (B) left ovary (lo).

**Figure 2:**
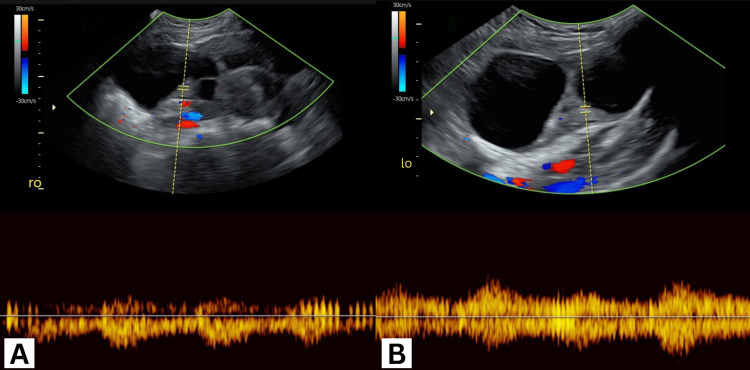
Doppler waveform demonstrating adequate blood flow to the; (A) right ovary (ro) and (B) left ovary (lo).

**Figure 3:**
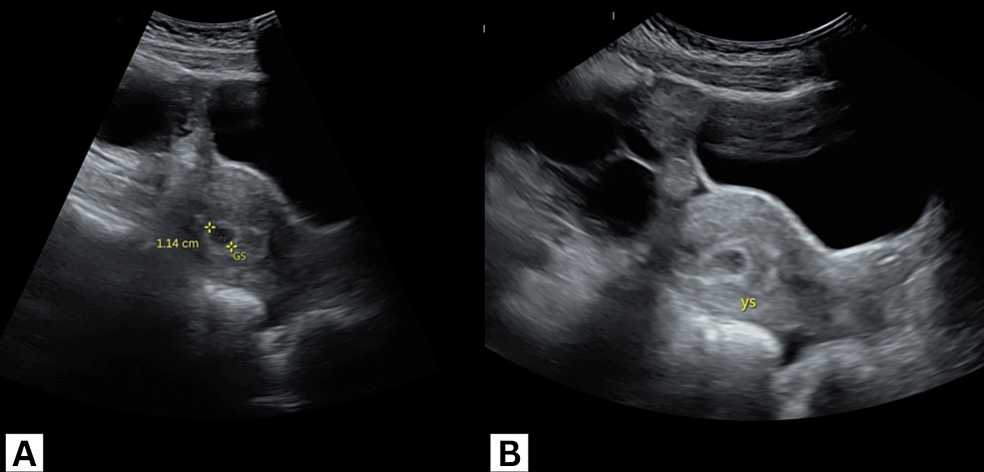
Transabdominal ultrasound images showing (A) gestational sac (GS) and (B) yolk sac (YS).

A transabdominal ultrasound showed bilateral enlarged multicystic ovaries measuring 10×5 cm on the right and 9×4.6 cm on the left (Figures 1A, 1B) with no signs of vascular compromise on either side (Figures 2A, 2B) or free fluid in the cul-de-sac. An intrauterine gestational sac measuring 11 mm and a yolk sac could be appreciated with no identifiable fetal pole (Figures 3A, 3B).

**Figure 4:**
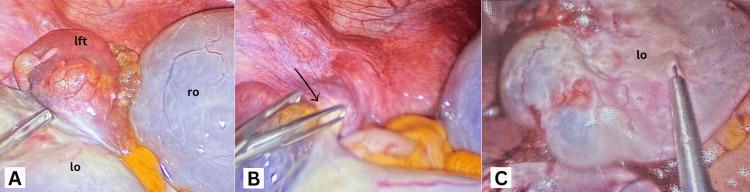
Laparoscopic images showing (A) the torted left fallopian tube and ovary, (B) left adnexa after 360-degree detorsion (black arrow shows the site of torsion), and (C) left ovary after cystic fluid aspiration. lo: left ovary, ro: right ovary, lft: left fallopian tube

The patient was admitted as a case of moderate recurrent OHSS for analgesia, IV fluids, and potassium infusion, and she was kept nil per os (NPO) with regular monitoring of vital signs. Over the next few hours, her symptoms worsened despite an initial improvement with conservative management. A repeat physical examination showed the abdominal mass larger than before, measuring about 10 cm in diameter and extending to the level of the umbilicus. An emergency laparoscopic surgery was done, and the suspected diagnosis of left-sided adnexal torsion was confirmed intraoperatively (Figure 4A). The ovary and fallopian tube, which were twisted twice around their own axis, were successfully detorted, and restoration of ovarian blood flow was confirmed by returning of visible pulsation (Figure 4B). A total of 200cc of straw-colored serous fluid was aspirated from multiple cysts bilaterally, and the surgery was completed with plication of the left ovarian ligament to prevent a recurrence (Figure 4C).

The patient recovered well postoperatively with major relief of symptoms. She was discharged two days later with a follow-up appointment in the early pregnancy assessment unit (EPAU) for further management.

## Discussion

Ovarian hyperstimulation syndrome (OHSS) is a condition characterized by an exaggerated stimulation of the ovaries following ovulation induction during assisted reproductive technologies and rarely due to mutation of the follicle-stimulating hormone receptors [6]. The incidence of moderate or severe OHSS after IVF treatments ranges from 3% to 6% and 0.1% to 2%, respectively, from which 16% develop ovarian torsion as a complication [7,8].

The exact pathophysiology of OHSS is not widely understood but is hypothesized to be related to hCG-mediated hypersensitivity of the ovaries [2]. This can manifest as multiple follicular and theca lutein ovarian cysts that are the leading cause of adnexal torsion, a condition characterized by twisting of the adnexa, ovaries, or fallopian tubes around its central axis formed by the infundibulopelvic and tubo-ovarian ligaments [8,9]. The impaired blood flow to the ovary causes stromal edema, hemorrhagic infarction, and necrosis [9].

There are multiple, well-established risk factors for the development of OHSS, which are classified as primary and secondary. Age less than 33 years, a low BMI, high antral follicle count (AFC), anti-mullerian hormone levels, and a previous history of OHSS or polycystic ovarian syndrome (PCOS) are some of the main primary risk factors. Factors associated with ovarian response to hormonal therapy including the number of ovarian follicles on the day of hCG administration, a high absolute level or rate of serum estradiol increase, elevated inhibin-B, and vascular endothelial growth factor (VEGF) are the most reported secondary risk factors [2].

OHSS is a clinical diagnosis with key findings of recent ovarian stimulation therapy followed by ovulation or hCG administration, symptoms of abdominal bloating, pain and nausea, decreased urine output, and a palpable abdominal mass. Pregnant women presenting with the same require an urgent transvaginal ultrasound (TVUS) scan or transabdominal scan (TAS), the imaging modality of choice in suspected cases of adnexal torsion [10]. A unilateral enlarged ovary and a coexisting mass, uniform peripheral cystic structures with or without intracystic hemorrhage, free fluid in the pelvis, and impaired arterial or venous flow with a twisted vascular pedicle are the most common ultrasound features seen in torsion [10]. Differential diagnoses such as ectopic pregnancy, ruptured ovarian cyst, tubo-ovarian abscess, and appendicitis among others should be considered and ruled out by carrying out relevant investigations including a complete blood count, beta-hCG levels, urea, electrolytes, and inflammatory markers [3]. Further management is guided by the severity of OHSS, classified by the Royal College of Obstetricians and Gynecologists [11].

Most cases of OHSS are self-limiting and require only supportive treatment with outpatient follow-up. Conservative treatment with analgesia and antiemetics, fluid and electrolyte replacement, and inpatient monitoring may be required in moderate-to-severe cases of OHSS as it can lead to life-threatening complications requiring surgical management as in this case. In high-risk cases, preventive measures such as cabergoline, prophylactic albumin or high-protein diet, metformin, low-dose aspirin, and relcovaptan among other strategies can be adopted to reduce the incidence of OHSS [11]. This patient had been managed with cabergoline after her diagnosis of OHSS, the effectiveness of which is described in several studies including randomized controlled trials and case reports [12,13].

Early surgical intervention is necessary to prevent patient morbidity including permanent ovarian damage and fetal loss associated with torsion [14]. Direct visualization of the torsion intraoperatively is the definitive diagnostic tool, and vascular assessment of the ovary guides the surgeon in the choice of the procedure from de torsion and ovary-sparing with or without oophoropexy or plication of the ovarian ligament, salpingo-oophorectomy, and cystectomy. Although surgeries are better avoided in pregnancy, laparoscopic detorsion is safe to be performed in the first trimester of pregnancy as done in this case [15,16]. Successful management of torsion associated with OHSS using this surgical option is reported in several case reports [17]. With this patient, the return of ovarian blood supply was intraoperatively confirmed by direct observation of ovarian pulsation, and the ovary was deemed safe to be spared.

Despite adequate treatment, the recurrence of torsion in pregnancy can be up to 19.5%, out of whom 73% conceived through assisted reproductive technologies. Seven out of 41 pregnant women had a recurrence of torsion in the same pregnancy [1].

## Conclusions

A history of ovulation induction therapy followed by IVF treatment, low BMI, and a previous history of OHSS were risk factors for the development of OHSS in the case presented above. Measures including prompt assessment of predisposing factors, serial ultrasound scans during ovulation induction and hormonal therapy in assisted reproductive techniques, and prophylactic cyst aspiration in confirmed cases of OHSS might reduce the prevalence of torsion and related complications. Further studies and extensive research are required to analyze the effectiveness of these management options.
